# Common and distinct morphometric correlates of the Dark Triad traits: machiavellianism, narcissism and psychopathy in a healthy male sample

**DOI:** 10.1007/s00702-026-03147-7

**Published:** 2026-04-06

**Authors:** Emilia L. Mielke, Corinne Neukel, Corinna Roth, Katja Bertsch, Friederike Nüssel, Sabine C. Herpertz

**Affiliations:** 1 Department of Psychiatry and Psychotherapy, Department of Psychosocial Medicine, Prevention, and Family Health, Medical Faculty, University of Heidelberg, University Hospital, Voßstraße 4, 69115 Heidelberg, Germany; 2 Department of Psychology, Julius-Maximilians-Universität Würzburg, Wuerzburg, Germany; 3 Ecumenical Institute, Faculty of Theology, University of Heidelberg, 69117 Heidelberg, Germany; 4 German Center for Mental Health (DZPG), partner site Mannheim/Heidelberg/Ulm, Mannheim, Germany

**Keywords:** Dark factor of personality, Personality disorder, Antisocial, Emotion recognition, Emotional intelligence, Prosocial

## Abstract

There is an ongoing debate about the common and distinct nature of the Dark Triad (DT) and its traits machiavellianism, narcissism, and psychopathy. Up to date, neurobiological data regarding this discussion has been scarce. We collected T1-weighted high-resolution structural magnetic resonance brain images from 24 healthy male subjects with high DT scores and 27 with low DT scores. Images were analyzed with voxel-based morphometry, compared between the two groups and correlated with DT subscales for machiavellianism, narcissism and psychopathy within the group with high DT scores. The group comparison revealed reduced gray matter volumes (GMV) in the precentral gyrus extending to the dorsolateral frontal gyrus, and the cerebellar crus II in individuals with high DT scores compared to those with low scores. In the regression analysis, we found negative associations between GMV in the dorsolateral prefrontal cortex and both machiavellianism and psychopathy. In addition, GMV in the anterior cingulate cortex was negatively associated with psychopathy and narcissism. Furthermore, GMV in the medial orbitofrontal cortex, insula, and superior temporal sulcus showed negative associations with narcissism. Our study revealed both common, overlapping as well as distinct GMV alterations related to DT traits. GMV alterations were primarily found in regions associated with empathy and social cognition. These structural differences may reflect neural substrates contributing to interpersonal dysfunction and antisocial tendencies observed in individuals with elevated DT traits.

## Introduction

Machiavellianism, subclinical narcissism and subclinical psychopathy are often subsumed under the same construct called the dark triad (DT) due to their overlap in socially malevolent behaviors and thoughts with tendencies toward emotional coldness, self-promotion, duplicity, and aggressiveness (Paulhus and Williams 2002). While DT traits are generally regarded as subclinical, as they do not necessarily meet the criteria for mental disorders or personality disorders, the behaviors associated with them can still lead to negative consequences for both the individuals themselves and those around them. It has been suggested to not differentiate between these three traits due to their common core of aversive traits. This has been conceptualized as the dark factor of personality. Individuals with high levels of the dark factor of personality are described as tending to maximize their own utility while either “disregarding, accepting, or malevolently provoking disutility for others - accompanied by beliefs that serve as justifications” (Moshagen et al. 2018, p. 657). Bader and colleagues (2022) found evidence supporting the concept of the dark factor of personality, as it was highly saturated with the aversive aspects of DT traits in two studies (total *n* > 1,000). However, narcissism and psychopathy displayed distinct features beyond the dark factor of personality. Nevertheless, since subdimensions of narcissism and psychopathy showed stronger correlations with facets of other DT traits than within their own construct, the authors argued for the existence of a single underlying dark factor of personality (Bader et al. 2022). In contrast, meta-analytical evidence suggests that while DT traits are positively related, they remain sufficiently distinct to warrant theoretical and empirical separation (O’Boyle et al. 2012). To date, it remains unclear whether there is one common dark factor of personality or whether machiavellianism, subclinical narcissism and subclinical psychopathy should be distinguished. As current evidence is largely based on self-report data and psychometric modeling, it remains an open question whether these constructs also share or differ in their neurobiological correlates. One approach to contributing to this question could be to study similarities and differences in brain morphometry associated with these traits. A few studies have investigated alterations in brain morphometry in relation to individual differences in machiavellianism, psychopathy, and narcissism separately. To the best of our knowledge, only one study has examined brain morphometry in relation to all three DT traits within a single sample.

Myznikov et al. investigated morphometric differences in relation to DT in healthy volunteers using a clustering approach, which resulted in a population-based comparison between individuals with “mid-to-high DT levels” and those with “low-to-mid DT levels. In this comparison, the “mid-to-high DT level” group showed reduced gray matter volume (GMV) in prefrontal regions (medial and dorsolateral prefrontal cortex, orbitofrontal cortex), the basal ganglia (nucleus accumbens, putamen, left caudate), the middle cingulate cortex and the right postcentral gyrus. The clustering was based on overall DT scores, and no analyses were conducted to examine the effects of individual DT traits separately (Myznikov et al. 2023).

However, the vast majority of previous studies have focused on morphometric correlates of DT traits separately. Verbeke et al. reported larger GMV in prefrontal clusters, including the medial, orbital, and dorsolateral prefrontal cortices, and in the insula in healthy sales professionals scoring high vs. low on machiavellianism (Verbeke et al. 2011). Another study reported a positive association between machiavellianism and GMV in the superior frontal gyrus, which was linked to social aggression in undergraduate students (Gong et al. 2022).

Nenadić et al. (2021) investigated correlations between narcissistic traits and brain morphometry in healthy subjects and discovered positive associations between narcissistic traits and GMV in prefrontal clusters, more specific in bilateral medial, orbital, and dorsolateral prefrontal cortices, as well as in the left insula (Nenadić et al. 2021). In contrast, Schmidt et al. reported a negative association between GMV in the insula and subclinical narcissism in healthy individuals (Schmidt et al. 2025). This finding is consistent with studies investigating brain morphometry in patients with narcissistic personality disorder, which have reported reduced GMV in the anterior insula and prefrontal regions (medial and dorsolateral prefrontal cortex, middle frontal gyrus), as well as the anterior cingulate cortex and right superior temporal cortex (Nenadic et al. 2015; Schulze et al. 2013).

For psychopathy, a voxel-based meta-analysis revealed reduced GMV in the left dorsolateral prefrontal and medial orbitofrontal cortex in individuals with psychopathic traits exceeding a clinical cutoff, as assessed by the Psychopathy Checklist. When psychopathy was further differentiated into two dimensions - Factor 1 (callous affect and interpersonal manipulation) and Factor 2 (impulsivity and antisocial behavior) - higher scores in both factors were associated with reduced GMV in the same prefrontal regions. Notably, several of the included studies were conducted in samples of criminal offenders (De Brito et al. 2021). Other studies, conducted either in forensic samples or in psychiatric populations, have also identified reduced GMV in the orbitofrontal cortex, the posterior cingulate cortex, the insula, superior temporal sulcus, and temporal pole region in relation to higher psychopathy (de Oliveira-Souza et al. 2008; Ermer et al. 2012). Studies investigating differences in GMV in subclinical psychopathy have been scarce. Chester et al. found a positive association between Factor 1 (see above) and GMV in the right anterior cingulate cortex and the amygdala in a mixed-sex sample of well-functioning adults. However, diagnoses of mental disorders were not assessed (Chester et al. 2023). Nummenmaa et al. investigated gray matter density (GMD) in both a healthy mixed-sex community sample as well as convicted violent male offenders with high psychopathic traits. Both samples demonstrated an association between higher Factor 1 psychopathic traits (see above) and lower GMD in the insula and orbitofrontal cortex (Nummenmaa et al. 2021).

In summary, common findings across previous studies on morphometric correlates of DT traits point to GMV alterations in prefrontal regions - particularly in the orbital and dorsolateral prefrontal cortex - as well as in the insula. Additionally, GMV alterations in the medial prefrontal cortex have been reported for both machiavellianism and narcissism, whereas changes in the anterior cingulate cortex and the superior temporal cortex have been observed in association with both narcissism and psychopathy. However, there is considerable heterogeneity across studies, even when distinguishing between clinical and subclinical populations.

The prefrontal cortex is known to play a crucial role in a variety of social, affective, and cognitive functions. Together with other regions like the superior temporal sulcus region, it is involved in mentalizing other peoples’ emotions, thoughts, and intentions (theory of mind; Molenberghs et al. 2016). Moreover, it contributes to the integration of information from brain areas involved in affective empathy, such as the insula and the anterior cingulate cortex (Jean Decety 2011). With respect to DT traits, a previous meta-analysis showed that higher narcissism and psychopathy were associated with reduced affective empathy, while higher Machiavellianism was linked to both reduced cognitive and affective empathy (Shukla and Upadhyay 2025). In fact, less emotional empathy in patients with narcissistic personality disorder has been associated with reduced GMV in the insula (Schulze et al. 2013). Therefore, the previously found structural alterations in individuals with machiavellianism, (subclinical) narcissism and (subclinical) psychopathy could translate to known deficits in empathic functions. Yet, findings to date are highly heterogeneous—both in terms of the traits studied and the direction and location of observed morphometric effects. Moreover, it remains an open question whether these traits share common neural correlates or reflect distinct neurobiological profiles.

To address this gap, the present study investigated both shared and unique patterns of GMV alterations associated with DT and its traits machiavellianism, subclinical narcissism and subclinical psychopathy. We focused on a sample of healthy male individuals in order to examine trait-related neural variation in the absence of clinical or forensic confounds, which often complicate the interpretation of previous findings. To identify common GMV alterations of DT, we first compared structural MRI data between individuals with high versus low DT personality traits. In a second step, we assessed trait-specific neural correlates by conducting a multiple regression analysis within the high DT group. All three DT traits were entered simultaneously into a single multiple regression model, allowing us to examine the unique contribution of each trait while controlling for the shared variance between them.

Based on prior literature, we expected GMV alterations in brain regions previously linked to DT traits and related socio-affective functions, including the dorsolateral prefrontal cortex, orbitofrontal cortex, medial prefrontal cortex, insula, anterior cingulate cortex, and superior temporal sulcus. We anticipated that these regions might show differences in both the group comparison (high vs. low DT) and the regression analysis, reflecting their potential role in shared as well as distinct aspects of DT. However, given the heterogeneity and inconsistency of previous findings, we refrained from formulating specific directional hypotheses or assigning particular regions to specific traits.

## Method

### Participants and measures

25 men with high DT personality traits (DT+) and 27 men with low DT personality traits (DT-) participated in the study. One data set had to be excluded from the analysis due to poor data quality of the MRI as indicated by extreme values and artifacts in the CAT12 “check data quality” module. Therefore, the final data set for the MRI study included 24 men with high DT personality traits (*M*_*age*_=22.8, *SD* = 2.7, range: 19–29 years) and 27 men with low DT personality traits (*M*_*age*_=26.7, *SD* = 6.3, range: 19–42 years).

Participants were recruited via local flyers and online advertisements in the Heidelberg area. Initially, an online screening survey was conducted in the general population to assess individual scores on DT traits using the Short Dark Triad (SD3; Jones and Paulhus 2014). The SD3 is a 27-item self-report instrument comprising three nine-item subscales measuring machiavellianism, narcissism, and psychopathy. Items are rated on a 5-point Likert scale ranging from strong disagreement to strong agreement. The scale was developed as a brief measure of subclinical DT traits and has demonstrated satisfactory internal consistency and construct validity in both community and student samples. In the original validation studies, mean scores in community samples ranged around 3.1–3.4 for machiavellianism, 2.8–3.0 for narcissism, and 2.3–2.4 for psychopathy for male participants (Jones and Paulhus 2014). In the present sample, internal consistency was high (Cronbach’s α = 0.946 for the total score; machiavellianism α = 0.895; narcissism α = 0.857; psychopathy α = 0.884).

A total of 264 individuals completed this online assessment. Based on their SD3 scores, participants scoring in either the lowest or highest quartile of the sample distribution were selected for potential inclusion in the MRI study. Inclusion criteria were: male sex, age between 18 and 65 years, right-handedness, native German language proficiency, and a score in the lower or upper quartile of the SD3 scale. Willingness to participate and undergo MRI measurement was also required. Selected individuals were contacted for a telephone screening. During this interview, exclusion criteria were assessed, including: current Axis I psychiatric disorder (as assessed via the Structured Clinical Interview for DSM-IV Axis I Disorders; SCID-I; First et al. 2002), lifetime diagnosis of schizophrenia, bipolar disorder, or neurodegenerative disease, regular intake of psychopharmacological medication, a history of traumatic brain injury, severe neurological or internal medical conditions, and any contraindications for MRI (e.g., metal implants, claustrophobia). Additionally, to exclude undiagnosed personality disorders, avoidant, narcissistic, antisocial, and borderline personality disorders were assessed by means of the semistructured International Personality Disorder Examination Interview (IPDE; Mombour et al. 1996). None of the participants met diagnostic criteria for a personality disorder.

The study was performed in accordance to the ethical standards laid out by the Declaration of Helsinki and approved by the local ethics committee of the Medical Faculty of the University of Heidelberg. All participants gave written consent before their participation after the study procedures were fully explained to them and received a monetary compensation.

### Structural MRI data

#### MRI data acquisition

We obtained a three-dimensional (3D) sagittal isotropic magnetization prepared rapid acquisition gradient echo (MPRAGE) sequence using a 3-Tesla MRI scanner (Tim Trio; Siemens, Erlangen, Germany) equipped with a 32-channel standard head coil (flip angle 9°, repetition time [TR] 1900ms, echo time [TE] 2.52ms, field of view 256 mm, matrix size 256 × 256 pixels, slice thickness 1 mm); 192 slices with an isotropic voxel size of 1 × 1 × 1mm3 were acquired. Experienced neuroradiologists reviewed all MRI scans to exclude clinically important abnormalities. In addition to the collection of structural data, a script-driven imagery paradigm was used to investigate protected and non-protected moral values (not reported here; Ueltzhöffer et al. 2023).

#### MRI data processing

Data was preprocessed using SPM12 (Statistical Parametric Mapping, Wellcome Trust Centre for Neuroimaging; London; UK; http://www.fil.ion.ucl.ac.uk/spm/) and the implemented CAT12-toolbox (Computational Anatomy Toolbox; https://neuro-jena.github.io/cat/) incorporated in MATLAB (Matrix Laboratory, The Mathworks: http://www.mathworks.de/products/matlab/). Unless stated otherwise, all operations were performed using CAT12 default settings for VBM (voxel-based morphometry) analyses. Preprocessing included the following steps: Normalization and segmentation into grey matter (GM), white matter (WM), and CSF, Estimation of total intracranial volume (TIV), quality and homogeneity checks as well as smoothing with an 8 mm full-width at half-maximum Gaussian kernel. The resulting files were then used for statistical analyses.

### Statistical analyses

Demographic and psychometric data of the groups with high and low DT personality traits were compared using independent-samples t-tests (IBM SPSS version 22.0). Group differences in structural MRI data were also assessed using a two-sample t-test. To examine associations between GMV and DT personality traits - machiavellianism, subclinical narcissism, and subclinical psychopathy - we performed a multiple regression analysis within the group with high DT personality traits. All three traits were included as regressors in a single multiple regression model to assess the unique effect of each trait while controlling for the others. The group with high DT personality traits was selected for this analysis due to its greater variance in SD3 subscale scores compared to the group with low DT traits. For both analyses (t-test, multiple regression), age and total intracranial volume (TIV) were included as covariates of no interest.

Both analyses were conducted using two complementary approaches: (1) a theory-driven region-of-interest (ROI) analysis using small volume correction (SVC), and (2) an exploratory whole-brain analysis. (1) In line with our theoretical framework and regions identified in previous research, a ROI mask was created using the Wake Forest University (WFU) Pickatlas based on the Automated Anatomical Labeling (AAL) Atlas. The mask included the bilateral dorsolateral prefrontal cortex (located in the superior and middle frontal gyrus, AAL Atlas), orbitofrontal cortex, medial prefrontal cortex, insula, anterior cingulate cortex and superior temporal sulcus. SVC was applied with a threshold of *p* <.05, family wise error (FWE) corrected for multiple comparisons within the ROI. (2) Both the t-test and multiple regression analyses were complemented by an exploratory whole-brain analysis to detect potential GMV alterations beyond the predefined regions. For this, statistical significance was set at *p* <.001 (uncorrected), with a minimum cluster size of k = 10 contiguous voxels. Anatomical labels for significant clusters were derived using the “cat12_aal3” atlas in the CAT12 toolbox.

## Results

### Demographic and psychometric data

Details on demographic and psychometric data can be found in Table 1. There was a significant group difference in age (*t*(35.84) = 3.01, *p*=.005, variance homogeneity could not be assumed). On average, the group with high DT personality traits was significantly younger than the group with low DT personality traits. Age was therefore added as a covariate in the statistical analyses of the structural MRI data (see 2.3). As expected based on our recruitment strategy, the group with high DT personality traits scored significantly higher on the SD3 total score, as well as on each of the subscales for machiavellianism, narcissism, and psychopathy (all *p* <.001).

**Table 1:** Demographic and psychometric data

	Group		Group comparison	
	DT+ (*N* = 24) *M* ± *SD*	DT- (*N* = 27) *M* ± *SD*	*t*	*p*
Age	22.8 ± 2.7	26.7 ± 6.3	3.01	0.005
Years of education	13.3 ± 0.7	13.4 ± 1.3	0.69	0.495
SD3 *overall Score*	3.3 ± 0.2	1.9 ± 0.1	−26.51	< 0.001
SD3 *machiavellianism*	3.6 ± 0.5	2.1 ± 0.4	−13.67	< 0.001
SD3 *narcissism*	3.7 ± 0.5	2.4 ± 0.4	−9.89	< 0.001
SD3 *psychopathy*	2.7 ± 0.5	1.3 ± 0.1	−13.54	< 0.001

*DT +*: group with high DT personality traits; *DT-:* group with low DT personality traits; *SD3:* Short Dark Triad, mean scores

### Structural MRI data

#### Group differences in structural MRI in individuals with high vs. low DT personality traits

Both the hypothesis-driven ROI analysis and the exploratory whole-brain analysis revealed larger GMV in the right precentral gyrus (46,3% of the cluster) extending to the superior dorsolateral frontal gyrus (17,0%), the supplementary motor area (14,3%) and the paracentral lobule (14,1%) (peak voxel MNI [−14, −17, 72], *k* = 368, *p*_*SVC*_=0.025) in individuals with low DT personality traits compared with individuals with high DT personality traits. Additionally, the exploratory voxel-based whole brain analysis revealed larger GMV in individuals with low DT personality traits compared with individuals with high DT personality traits in a cluster located in the right Crus II extending to Crus I of the cerebellar hemisphere. Conversely, individuals with high DT personality traits compared to individuals with low DT personality showed larger GMV in bilateral clusters of the inferior occipital gyrus (see Table 2).

**Table 2:** MNI coordinates and anatomical locations of peak effects of the voxel-based analysis in the direct group comparison

Contrast	Cluster size k	t(47)	*p*_uncorr_	Peak voxel MNI: x,y, z (mm)	Anatomical location of peak voxels (cat12_aal3)
DT + < DT-	637	5.23	< 0.001	18, −20, 68	Rt. Precentral G.
	77	3.9	< 0.001	38, −50, −42	Rt. Crus II of Cerebellar Hemisphere
DT + > DT-	220	4.47	< 0.001	−38, −86, −8	Lt. Inferior Occipital G.
	29	3.72	< 0.001	48, −81, −15	Rt. Inferior Occipital G.

*DT +:* group with high Dark Triad (DT) personality traits; *DT-:* group with low DT personality traits

*cat12_aal3* = Automated Anatomical Labeling (AAL) Atlas of the CAT12-toolbox (Computational Anatomy Toolbox)

#### Multiple regression analysis:associations of structural MRI and machiavellianism, subclinical narcissism, and subclinical psychopathy in the group with high DT personality traits

*Machiavellianism.* Both the hypothesis-driven ROI analysis and the exploratory whole-brain analysis revealed a significant negative association between machiavellianism and GMV in the left superior dorsolateral prefrontal cortex (peak voxel MNI [−21, 33, 30], *k* = 496, *p*_*SVC*_=0.015). The exploratory voxel-based whole brain analysis revealed significant positive associations between machiavellianism and GMV in clusters located in the right temporal pole, the right precuneus, the left middle cingulate gyrus, and the right lingual gyrus. Significant negative associations between machiavellianism and GMV were found in clusters located in the left parahippocampal gyrus, the right Crus I extending to Crus II of the cerebellar hemisphere, and the left hippocampus (see Table 3).

*Subclinical narcissism.* The hypothesis-driven ROI analysis revealed no significant associations between subclinical narcissism and GMV in the a-priori defined regions. The exploratory voxel-based whole brain analysis revealed a significant positive association between subclinical narcissism and GMV in a cluster located in the right calcarine gyrus. Significant negative associations between subclinical narcissism and GMV were found in clusters located in the bilateral anterior cingulate cortex, the left parahippocampal gyrus, the bilateral medial orbitofrontal gyrus, the right superior temporal gyrus and the right insula extending to the right temporal pole (see Table 3).

*Subclinical psychopathy.* The hypothesis-driven ROI analysis revealed no significant associations between subclinical psychopathy and GMV in the a-priori defined regions. The exploratory voxel-based whole brain analysis revealed significant positive associations between subclinical psychopathy and GMV in clusters located in the left fusiform gyrus, the inferior temporal gyrus and the left Lobule IV-V of the cerebellar hemisphere. Significant negative associations between subclinical psychopathy and GMV were found in clusters located in the bilateral anterior cingulate cortex and the left dorsolateral superior frontal gyrus (see Table 3).

**Table 3:** MNI coordinates and anatomical locations of peak effects of the voxel-based analysis in the multiple regression analysis in the group scoring high on DT

Contrast		Cluster size k	t(18)	*p*_uncorr_	Peak voxel MNI: x,y, z (mm)	Anatomical location of peak voxels (cat12_aal3)
machiavellianism x GMV	positive association	139	4.63	< 0.001	30, 15, −33	Rt. Temporal Pole
		17	4.47	< 0.001	14, −45, 45	Rt. Precuneus
		51	4.09	< 0.001	−9, −21, 39	Lt. Middle Cingulate G.
		13	3.94	< 0.001	14, −93, −5	Rt. Lingual G.
	negative association	87	6.71	< 0.001	−21, 33, 30	Lt. Dorsolateral Superior Frontal G.
		115	4.68	< 0.001	−24, −6, −38	Lt. Parahippocampal G.
		298	4.42	< 0.001	42, −68, −38	Rt. Crus I of Cerebellar Hemisphere
		73	3.97	< 0.001	−35, −20, −11	Lt. Hippocampus
subclinical narcissism x GMV	positive association	22	4.28	< 0.001	24, −71, 6	Rt. Calcarine G.
	negative association	110	5.43	< 0.001	0, 32, 29	Bil. Anterior Cingulate Cortex
		413	5.27	< 0.001	−24, −6, −38	Lt. Parahippocampal G.
		450	4.94	< 0.001	−14, 47, −24	Lt. Medial Orbitofrontal G.
		72	4.57	< 0.001	72, −21, 2	Rt. Superior Temporal G.
		232	4.30	< 0.001	−9, 33, 12	Lt. Anterior Cingulate Cortex
		22	4.09	< 0.001	47, 9, −9	Rt. Insula
		38	4.00	< 0.001	14, 44, −24	Rt. Medial Orbitofrontal G.
subclinical psychopathy x GMV	positive association	241	4.87	< 0.001	−32, −68, −14	Lt. Fusiform G.
		14	4.67	< 0.001	−50, −30, −18	Lt. Inferior Temporal G.
		12	3.93	< 0.001	−17, −38, −20	Lt. Lobule IV-V of Cerebellar Hemisphere
	negative association	142	4.36	< 0.001	3, 41, 14	Bil. Anterior Cingulate Cortex
		23	4.08	< 0.001	−21, 59, 14	Lt. Dorsolateral Superior Frontal G.

*DT *: Dark Triad; *GMV:* Gray matter volume; *cat12_aal3:* Automated Anatomical Labeling (AAL) Atlas of the CAT12-toolbox (Computational Anatomy Toolbox)

## Discussion

In the current study, we used voxel-based morphometry to investigate common and distinct morphometric correlates of the DT traits machiavellianism, subclinical narcissism, and subclinical psychopathy. For the first time, this was done within a comprehensive design combining both a group comparison between individuals with high vs. low DT personality traits and a trait-specific multiple regression analysis assessing associations between GMV and the three DT traits. Importantly, although the group with high DT traits showed comparatively elevated SD3 scores relative to general population samples reported in the literature (Jones and Paulhus 2014), all participants were screened using structured clinical interviews and none met criteria for a personality disorder. Thus, the present findings reflect subclinical personality variation within a healthy male sample rather than clinically manifest personality pathology.

Our study revealed reduced GMV in the right precentral gyrus extending into the superior dorsolateral frontal gyrus in individuals with high DT personality traits compared to those with low DT traits. While the precentral gyrus is primarily involved in motor planning and execution, it has also been implicated in processes related to action observation and imitation. These mechanisms are thought to contribute to emotional empathy (Del Casale et al. 2017), as suggested by the action perception model, which posits that understanding others’ emotions partly relies on internally simulating their actions (de Waal and Preston 2017). Such simulation processes may be relevant for the recognition of emotional expressions and the ability to respond empathically. In line with this, a meta-analysis reported negative associations between DT traits - particularly psychopathy and machiavellianism - and emotional intelligence, which includes the ability to perceive and interpret others’ emotions (Miao et al. 2019). Altered GMV in the precentral region could thus be one contributing factor to the reduced emotional responsiveness often reported in individuals with high DT traits (Jonason and Krause 2013).

Although only a portion of the cluster (17%) extended into the dorsolateral prefrontal cortex, this is consistent with previous findings of reduced GMV in this region in individuals with high DT traits (Myznikov et al. 2023). The dorsolateral prefrontal cortex plays a central role in abstract reasoning and cognitive control over emotion – for example in moral decision-making tasks (Greene et al. 2004; Pascual et al. 2013). Larger GMV in this area has been associated with impartial behavior through perspective-taking (Baumgartner et al. 2013). Functional data from a related fMRI study conducted in the same sample (not reported here) also showed increased activation of the dorsolateral prefrontal cortex in response to appeals to protected versus non-protected moral values (Ueltzhöffer et al. 2023). Moreover, structural and functional alterations in this region have been linked to antisocial behavior in prior meta-analyses (Yang and Raine 2009), underscoring its potential relevance for traits associated with diminished moral and empathic functioning.

Our multiple regression analysis also revealed negative associations between GMV in the left dorsolateral prefrontal cortex and both machiavellianism, and subclinical psychopathy. This is in line with previous studies suggesting a link between frontal lobe dysfunction and higher machiavellianism in patients with Parkinson’s disease and delusional disorder (Brüne et al. 2010; McNamara et al. 2007). In addition, Cohen-Zimerman et al. discovered, that brain damage specific to the left dorsolateral prefrontal cortex was associated with higher machiavellianism (Cohen-Zimerman et al. 2017). Regarding psychopathy, a previous voxel-based meta-analysis and meta-regression found a negative association between psychopathy and GMV in the dorsolateral prefrontal cortex (De Brito et al. 2021). In their meta-analysis, De Brito et al. (2021) linked decreased GMV in this region to impairments in memory and social cognition. GMV reductions in the dorsolateral prefrontal cortex in individuals with high DT personality traits, especially machiavellianism and psychopathy, might therefore contribute to reduced moral concern and antisocial tendencies commonly associated with these traits.

The exploratory whole brain analysis of group differences in structural MRI also revealed smaller GMV in crus II of the cerebellar hemisphere in individuals with high vs. low DT personality traits. Previous studies have found altered function and reduced GMV of the cerebellum in patients with antisocial personality disorder and psychopaths (Moreno-Rius 2019). In psychopaths, reduced GMV of the posterior cerebellum has been found to be associated with deficits in facial emotion recognition capabilities (Pera-Guardiola et al. 2016). In fact, a meta-analysis of fMRI studies has linked the cerebellar crus II to mentalizing with most studies showing its involvement in emotion recognition through facial and bodily expression (Van Overwalle et al. 2020).

Interestingly, our multiple regression analysis revealed a positive association between machiavellianism and GMV in the temporal pole, which has been linked to moral decision-making (Heekeren et al. 2003). The temporal pole is also known to be involved in autobiographic memory function, emotion recognition and theory of mind (Herlin et al. 2021). Although results have been mixed, most studies found a negative association between machiavellianism and theory of mind capabilities (Al Aïn et al. 2013; Duradoni et al. 2023). One study provided preliminary evidence that emotionally manipulative strategies may be associated with high levels of machiavellianism specifically among individuals with strong emotion recognition abilities (Schmitt et al. 2020). Further research is needed to clarify the neural underpinnings of machiavellianism and their links to specific cognitive and emotional processes.

Subclinical narcissism was negatively associated with GMV in the bilateral anterior cingulate cortex, the bilateral medial orbitofrontal cortex, the superior temporal gyrus and the insula. This is in line with studies investigating GMV alterations in patients with narcissistic personality disorder (Nenadic et al. 2015; Schulze et al. 2013). However, our study consisted of individuals with subclinical narcissism and a previous study in this regard has found positive associations between narcissistic traits and GMV in the medial orbitofrontal cortex and the left insula (Nenadić et al. 2021). In contrast, Schmidt et al. reported a negative association between GMV in the insula and subclinical narcissism in healthy individuals (Schmidt et al. 2025). Nenadić et al. suggested that their differing results between subclinical and clinical narcissism might be explained by a non-linear inverted-U-shape association across a broader continuum (from nonclinical to pathology). Our study only consisted of male participants, who are known to have higher narcissistic traits compared to women (Grijalva et al. 2015), and we performed the multiple regression analysis only within the group with high DT personality traits. Assuming an inverted-U-shaped relation, it is possible that participants in our sample, who represent a high subclinical range, show negative associations between narcissism and GMV in these regions. We also need to consider that previous studies did not account for related factors, that could have explained variance in the identified brain structure. To the best of our knowledge, our study is the first to identify GMV alterations related to DT and its subscales in a comprehensive multiple regression analysis. Therefore, our results of negative associations between subclinical narcissism and GMV in the anterior cingulate cortex, the medial orbitofrontal cortex, the superior temporal gyrus and the insula reflect unique effects of narcissism after accounting for shared variance with machiavellianism and subclinical psychopathy.

Schulze et al. suggested that reduced GMV in the aforementioned brain regions might be related to impairments in empathy (Schulze et al. 2013), a hallmark feature of patients with narcissistic personality disorder (Ronningstam 2010). In fact, they found a positive association between emotional empathy and GMV in the anterior insula, which was reduced in patients with narcissistic personality disorder (Schulze et al. 2013). Together with the anterior insula, the anterior cingulate cortex has also been suggested as a key region of emotional empathy (Singer and Lamm 2009) and GMV reductions in both regions have been associated with reduced emotional empathy (Cerami et al. 2014; Eres et al. 2015). In fact, both regions have been previously suggested as key regions in a neural model of mechanisms of empathy deficits in narcissism (Jankowiak-Siuda and Zajkowski 2013). In line with a previous voxel-based meta-analysis and meta-regression, our study also revealed a negative association between subclinical psychopathy and GMV in the anterior cingulate cortex (De Brito et al. 2021). This could relate to the lack of empathy and prosocial behavior also seen in individuals with high psychopathic traits (Viding and McCrory 2019).

Our study also showed negative associations between subclinical narcissism and GMV in the medial orbitofrontal cortex and the superior temporal gyrus. The medial orbitofrontal cortex is known for its role in emotion-related learning by representation of reward or affective value of stimuli (Rolls 2019). Together with the amygdala and superior temporal gyrus, it is involved in the processing of emotion information (Decety 2010) and has therefore been suggested as another component of a broader network contributing to mechanisms of empathy deficits in narcissism (Jankowiak-Siuda and Zajkowski 2013).

### Limitations

The following limitations must be considered: (1) Our exploratory whole brain analysis revealed significant results in hypothesized regions, that did not survive correction for multiple comparison and therefore did not reach significance in the regions-of-interest analysis. Due to the lack of corrected significance and the relatively small sample size further studies are needed before firm conclusions can be drawn. (2) As we considered only a male sample, we cannot draw conclusions on sex-specific differences. (3) It remains unclear if and how structural brain alterations are linked to functional correlates as multiple factors contribute to structural volume such as the size of glial cells and neurons, vascularity and density. The relationship between structural and functional alterations remains debatable and should be further examined using fMRI and behavioral studies.

### Conclusion

Taken together, the current study revealed both common and distinct morphometric correlates of DT. Regarding common morphometric alterations, our group comparison showed smaller GMV in the right precentral gyrus extending to the superior dorsolateral frontal gyrus, and the cerebellar crus II in individuals with high DT personality traits compared with individuals with low DT personality traits. In addition, both machiavellianism and subclinical psychopathy were negatively associated with GMV in the left superior dorsolateral prefrontal cortex. These structural findings may reflect differences in brain regions previously associated with social and affective processes, although functional implications should be interpreted with caution. Alterations in the precentral gyrus and the cerebellar crus II might relate to processes such as emotion recognition, while common alterations in the dorsolateral prefrontal cortex could translate to a lacking concern for morality and antisocial behavior associated with these traits. Both subclinical psychopathy and subclinical narcissism, but not machiavellianism, were negatively associated with GMV in the anterior cingulate cortex, a region often discussed in the context of emotional empathy and social cognition. Only subclinical narcissism was also negatively associated with GMV in the bilateral medial orbitofrontal cortex, the superior temporal gyrus and the insula – regions, that have been previously discussed as a broader network contributing to mechanisms of empathy deficits in narcissism. Our study contributes to previous meta-analytical evidence showing that the DT traits are positively related to one another but sufficiently distinctive to justify theoretical and empirical separation (O’Boyle et al. 2012). Our study revealed both common GMV alterations of DT in the group comparison, as well as overlapping GMV alterations among some DT traits. Other GMV alterations, however, appear to be solely related to single DT traits. While interpretations of brain-behavior relationships must remain cautious, GMV alterations were primarily found in regions associated with empathy and social cognition. More research in this field could help to further illuminate common and distinct features of the dark triad traits machiavellianism, subclinical narcissism and subclinical psychopathy and how they relate to antisocial behavior and empathy deficits.

## Data Availability

The data that support the findings of this study are not publicly available due to ethical and legal restrictions. Data may be available from the corresponding author upon reasonable request and with permission of the local ethics committee.
